# Thermionic Energy Conversion Based on Graphene van der Waals Heterostructures

**DOI:** 10.1038/srep46211

**Published:** 2017-04-07

**Authors:** Shi-Jun Liang, Bo Liu, Wei Hu, Kun Zhou, L. K. Ang

**Affiliations:** 1 SUTD-MIT International Design Center (IDC), Singapore University of Technology and Design (SUTD), 8 Somapah road, 487372, Singapore; 2 School of Mechanical and Aerospace Engineering, Nanyang Technological University, 50 Nanyang Avenue, 639798, Singapore; 3 Computational Research Division, Lawrence Berkeley National Laboratory, Berkeley, CA 94720, USA

## Abstract

Seeking for thermoelectric (TE) materials with high figure of merit (or ZT), which can directly converts low-grade wasted heat (400 to 500 K) into electricity, has been a big challenge. Inspired by the concept of multilayer thermionic devices, we propose and design a solid-state thermionic devices (as a power generator or a refrigerator) in using van der Waals (vdW) heterostructure sandwiched between two graphene electrodes, to achieve high energy conversion efficiency in the temperature range of 400 to 500 K. The vdW heterostructure is composed of suitable multiple layers of transition metal dichalcogenides (TMDs), such as MoS_2_, MoSe_2_, WS_2_ and WSe_2_. From our calculations, WSe_2_ and MoSe_2_ are identified as two ideal TMDs (using the reported experimental material’s properties), which can harvest waste heat at 400 K with efficiencies about 7% to 8%. To our best knowledge, this design is the first in combining the advantages of graphene electrodes and TMDs to function as a thermionic-based device.

The most common approach to harvest the waste heat to generate electricity is thermoelectrics (TE), which is based on the Seebeck effect (see Table 1). The performance of TE-based devices is characterized by the figure of merit (*ZT*), given by ref. 1


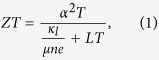


where *α, T, κ*_*l*_, *μ, n*, and *e* are, respectively, the Seebeck coefficient, absolute mean temperature, lattice thermal conductivity, carrier mobility, carrier density and electron charge. Here, *L* is defined as the Lorenz number equal to 2.44 × 10^−8^ WΩ K^−2^. This formula has recently been redefined to solve the inconsistence between theoretical predication and experimental measurement^2^. Before the 1990s, the progress of improving *ZT* had been slow and the best TE material was Bi_2_Te_3_ alloys with *ZT* ≈ 1.0 at 300 K^3^. To increase *ZT*, many new approaches have been proposed^4^,^5^, such as fabricating low-dimensional thermoelectric structures to increase large density of state, engineering the interface of materials to reduce the lattice thermal conductivity, and modulating dopants to increase carrier mobility. Subsequently, further improvements include *ZT* = 2.4 at 300 K for *p*-type *Bi*_2_*Te*_3_/*Sb*_2_*Te*_3_ superlattice^6^, and *ZT* = 3 at 550 K for Bi-doped n-type PbSeTe/PbTe quantum-dot superlattice^7^. A prospective of nanostructured TE materials can be found in a review paper^8^. For practical applications, other issue such as size, maintenance and fast response time must also be considered even if high-efficiency TE materials are found ref. 1.

Recent interests in using two-dimensional (2D) transition metal dichalcogenides (TMDs) as new TE materials have attracted extensive attention^9^,^10^ due to high Seebeck coefficient offered by these 2D TMDs: a bilayer MoS_2_ gives *α*^2^*σ* = 8.5 mW/m/K^2 ^9. If this MoS_2_-based TE material is able to realize the calculated thermal conductivity of *κ* ≈ 1.55 W/m/K^11^, we will have *ZT* = 1.6, which corresponds to an efficiency of about 6.5% in harvesting waste heat at *T*_*h*_ (hot side) = 400 K and *T*_*c*_ (cold side) = 300 K [according to Eq. (1)].

For high temperature range, the more viable approach is based on thermionic energy convertor (TIC), which was first proposed by G. N. Hatsopoulos^12^. Due to the high work function of the metallic electrode, however TIC is limited to high-temperature operation above 1500 K. A potential method to harvest waste energy at 900 K was recently proposed by using a suspended monolayer graphene as cathode to provide an efficiency of higher than 40%^13^. This improvement is attributed to the new thermionic law given by *J(ϕ, T, E*_*F*_) = A^*^ × *T*^3^ × *exp*[−(*eϕ* − *E*_*F*_)/*k*_*B*_*T*], where 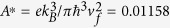 A/cm^2^/*K*^3^, *v*_*f*_ is the Fermi velocity, *E*_*F*_ is the Fermi level, and *ϕ* is the barrier height at zero bias. Note that the new scaling has been compared well with a recent experiment^14^. For the wasted heat generated in the industrial or domestic process, low-grade heat (around 400 K to 500 K) is distributed more everywhere. developing an efficient approach remains a great challenge so far.

In this paper, we propose a high-efficiency solid-state thermionic device by using van der Waals (vdW) heterostructure^15^ composed of 2D TMDs (MoS_2_, MoSe_2_, WS_2_, and WSe_2_) and graphene electrodes. By taking the advantage of the ultralow cross-plane thermal conductance of the 2D materials and the new thermionic emission over the Schottky barrier (SB) contact between the graphene and 2D materials (tunable via gate voltage or chemical doping), we predict that it is possible to realize high-efficiency power generation and refrigeration at the temperature of 300 K to 500 K, which may be better than (or at least comparable to) the traditional TE devices. Note that the concept of using multi-layers or superlattices in the thermionic devices was first suggested by two groups (Shakouri and Mahan) in late 1990s^16^,^17^. The performance of their proposed single-junction thermionic device was predicted to be better than the TE device using the same lnGaAs/lnAlAs material^18^. For simplicity, we will ignore the effect of non-conservation of lateral momentum in the thermionic emission^18^,^19^ in this paper. This is justified by the facts that Schottky barrier height is planar and homogenous at the interface between graphene and Transition metal dichalcogenide^20^.

With the current advances in growing graphene and TMDs, the proposed vdW heterostructures such as Gr/TMDs/Gr (Gr is the monolayer graphene) can be assembled experimentally^15^,^21^,^22^,^23^,^24^,^25^,^26^. For different 2D TMDs (MoS_2_, MoSe_2_, WS_2_ and WSe_2_), the cross-plane thermal conductivity *κ* was measured to be very low: *κ* = 0.05 W/m/K for disordered WSe_2_^27^, *κ* = 0.0084 to 0.3 W/m/K for MoS_2_, WS_2_ and WSe_2_^28^, and *κ* = 0.085 W/m/K for 10 nm-thickness of WSe_2_ synthesized via Se-O exchange^29^. Due to their low cross-plane thermal conductivity, these 2D materials may seem to be good TE materials, but having very low electrical mobility^30^ will also offset the increment due to low thermal conductivity. Thus the thermionic emission-based (or TIC) method may be a better approach than TE method, if the ballistic transport^31^ within the structure can be ensured by choosing a suitable thickness (to avoid collisions) and an optimal barrier height (to have high-current injection). Note that this ballistic assumption is very commonplace in the previous studies of thermionic emission based on the traditional Superlattices structure^17^,^18^,^32^. In the conventional superlattices, the thickness is usually larger then 1 *μ*m. Compared with the conventional suplattices structure, the van der waals heterostructure made of graphene and TMDs considered in our paper is far less than 1 *μ*m in thickness of cross-plane direction. Therefore the ballistic assumption in the cross-plane is justified. Note that Gr/h-BN/Gr heterostructures device^33^ has been fabricated to study the thermoelectric transport properties, but the measured low ZT calls for further effort to identify the most ideal sandwiched material and optimized parameters of devices to achieve the goal of high-efficiency thermal energy conversion. In our work presented here, we theoretically identify two materials WSe_2_ and MoSe_2_, together with employing Molecular dynamics simulations and ab initio calculations. we hope that these two identified candidates materials can further motivate experimentalist to explore the feasibility of achieving high-efficiency thermal energy harvesting system based on thermionic emission mechanism in using other 2D materials assembled in a vdW heterostructure.

Figure 1 illustrates the proposed vdW heterostructure-based thermionic device with two monolayers graphene as top and bottom layers, and *N*-layers of 2D TMDs materials of thickness *d* between two graphene layers. This configuration is similar to the recently-reported Gr/phosphorene/Gr^34^ and Gr/h-BN/Gr sandwich structure^33^. The device can be either a power generator or refrigerator depending on the direction of the current flow. For power generation, electron flow is from the hot electrode at temperature *T*_*h*_ to the cold electrode at temperature *T*_*c*_.

If a 2D material with suitable thickness *d* and low cross-plane thermal conductivity *κ* is used as the vdW heterostruture, our calculations show that TIC device may offer higher efficiency as compared to the existing TE devices operating at low-grade temperature (400 to 500 K). As a power generator, it can harvest waste heat at 400 K with about 8% efficiency using the reported experimental properties of the 2D materials.

For refrigeration at 260 K, the efficiency can be more than 40% of the Carnot efficiency. According to our model, the thickness of the layer must be in the intermediate range in order to reach high efficiency. For few layers, electron tunneling process will be dominant over the over-barrier thermionic emission which will reduce the efficiency^35^. For larger values of *d*, it will induce a large cross-plane thermal conductivity *κ*; and also makes the assumption of ballistic electron transport within the layers no longer valid.

For a given voltage of *V*, the electrical current density (*J*_*e*_) and the thermal current density (*J*_*Q*_) being transported across the electrodes are, respectively,


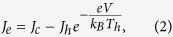



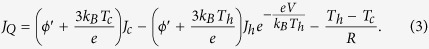


Here, 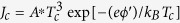
13 is the thermionic current density emitted from the cold graphene electrode at temperature *T*_*c*_ over an effective Schottky barrier height of *ϕ*′ formed at the interface between the vdW structure and the graphene electrode. Similarly, we have 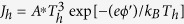 for the thermionic current density emitted from the hot graphene electrode at temperature *T*_*h*_. Due to the unique properties of graphene in tuning its Fermi energy via bias voltage or chemical doping, the effective barrier is defined as *ϕ*′ = *ϕ* − *E*_*F*_/*e*, where *ϕ* is the value at zero bias with an intrinsic Fermi level.

As already pointed out by previous work^13^,^36^, traditional vacuum thermionic converter cannot operate near room temperature due to the high vacuum work function (around 4.51 eV) of graphene. To operate at room temperature, the work function is required to be below 0.34 eV^36^, which can be attainable in the graphene/TMDs heterostructure. In Fig. 2, we present the verification (in comparison with experimental results) of the revised thermionic emission law^13^ is valid in describing the electron flow over the Schottky barrier of graphene/TMDs contact for graphene/MoSe_2_ (44 layers) contact^24^ with a reported barrier height of *ϕ*′ = 0.2 V (best fit). The calculated value of *ln(J*/*T *^3^) has an excellent agreement with experiment^24^ from 1000/*T* (about T = 250 to 300 K) as shown in Fig. 2a. The agreement with experiment indicates that our thermionic emission model can be good enough to describe the carrier’s transport across the Vdw heterostructure.

A prior first-principles calculation^37^ has also indicated that the conduction band edge of MoSe_2_ will reduce with increasing layer number (black line with black symbols in Fig. 2b). As a result, the Schottky barrier height decreases from 0.614 to 0.38 volt as the layer number increases from one layer to eight layers (red line with symbols) as shown in Fig. 2b. The trend of change in Schottky barrier height with increasing layer number is consistent with previous experiment^24^, which reports that the Schottky barrier height between graphene and MoSe_2_ becomes saturable with larger than 50 MoSe_2_ layers. Based on the experiment facts and first-principle calculation, it is reasonable to believe that the Schottky barrier height can be reduced down to around 0.2 volt when the layer number increases up to 44 layers. For completeness, the band structure of other contacts between graphene and one layer of TMDs materials (MoS_2_, WS_2_ and WSe_2_) can be found in the Supplementary materials (see S3).

In Eq. (3), the 3*k*_*B*_*T* term measures the average heat energy per emitted electron, which is obtained through the internal energy of electron in graphene associated with one degree of freedom, 
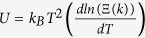
, with Ξ(*k*) being the partition function. In the last term of Eq. (3), *R* is the thermal resistance including all the contributions due to interface (between graphene and TMDs, and different layers within TMDs), barrier layers and electrode. We will only consider thermal conductance due to TMDs and limit our study to multi-layers TMDs, as the molecular dynamics simulation (see discussions section) shows that the contribution from the interface between graphene and TMDs and electrode is small, as compared to the resistance due to TMDs itself. For simplicity and a conservative estimation, we use *R* = *d*/*κ* in Eq. (3). Including other effects will increase the efficiency predicted in this paper. By defining the average temperature as *T* = (*T*_*h*_ + *T*_*c*_)/2, and the temperature difference as *δT* = *T*_*h*_ − *T*_*c*_, we calculate *J*_*e*_ as the thermionic emitted current density at the mean temperature *T*. In the limit of 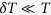 and 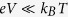, Eqs (2) and (3) become


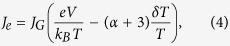



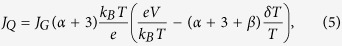


where *α* = *eϕ*′/*k*_*B*_*T*, 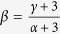, *γ* = (*T*_*R*_/*T*)^3^*e*^*α*^, and 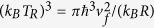. Here, we have introduced a temperature-like parameter *T*_*R*_ to characterize the performance of the device (together with the effective barrier height, *ϕ*′), where *T*_*R*_ is proportional to (*κ*/*d*)^1/3^ or inversely proportional to the barrier resistance, *R*^−1/3^. Numerically, we have *T*_*R*_ [K] = 4666 × (*κ*/*d*)^1/3^ for *κ* [W/m/K] and *d* [nm].

The experimental verification of our model proposed in this work closely depends on the design of Gr/TMDs/Gr. In this paragraph, we will briefly state that our design of Gr/TMDs/Gr can be easily realized by using the current capability in fabrication of vdW heterostructure by many different research groups^22^,^23^,^24^. For example, Gr/WSe_2_/Gr had been fabricated ranging from *d* = 2.2 nm (3 layers of WSe_2_) to 40 nm^22^. Similarly, we have *d* = 50 nm and *d* = 9 to 50 nm, respectively, for Gr/MoS_2_/Gr^23^, and for Gr/MoSe_2_/Gr^24^. The combination of more than one type of TMDs materials is also possible, such as Gr/MoS_2_/WSe_2_/Gr^38^. Thus we have used some of reported experimental parameters to illustrate the performance of our design in harvesting waste heat at 400 K as shown below.

## Results

### Refrigerator

For the device to operate as a refrigerator, it require *J*_*Q*_ > 0, which poses a condition of 
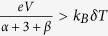
 in Eq. (5). The cooling efficiency is calculated by *η* = *J*_*Q*_/(*J*_*e*_ × *V*) and its maximum efficiency (*η*_*max*_) is obtained by taking first derivative with respect to *V*, which gives


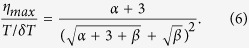


**Figure 3:**
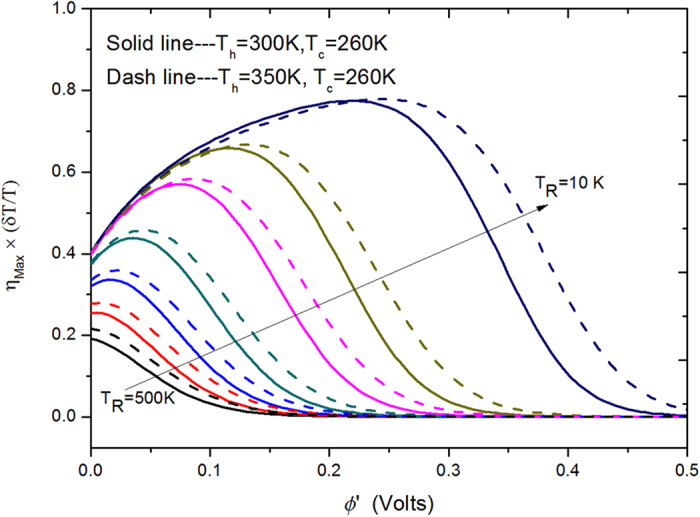
The maximum cooling efficiency *η*_*max*_ (in terms of the Carnot efficiency) as a function of barrier height *ϕ*′ at *T*_*c*_ = 260 K for *T*_*h*_ = 300 K (solid lines) and 350 K (dashed lines) from *T*_*R*_ [K] = 500, 400, 300, 200, 100, 50 and 10 (according to the arrow direction).

The variables *α* and *β* are functions of *ϕ*′ and *T*_*R*_. The term *T*/*δT* is approximately regarded as the Carnot efficiency. In Fig. 3, we plot the maximum cooling efficiency *η*_*max*_ (in terms of the Carnot efficiency) at *T*_*c*_ = 260 K as a function of effection barrier height *ϕ*′ = 0 to 0.5 volt for various *T*_*R*_ = 500 K down to 10 K at *T*_*h*_ = 300 K (solid lines) and 350 K (dashed lines). It is clear that the cooling efficiency increases with smaller *T*_*R*_ as expected. For a given *T*_*R*_, the maximal efficiency can be achieved by tuning the effective barrier height *ϕ*′. The tuning range will become wider for higher temperature (e.g. *T*_*h*_ = 350 K) as compared to *T*_*h*_ = 300 K. In the limit of *T*_*R*_ = 0, we have *γ* = 0, 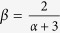, and Eq. (6) becomes 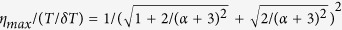, which depends only on *α* (or *ϕ*′).

With few reported values of the cross-plane thermal conductivity for vdW heterostructure, we use two measurements (at 300 K)^27^,^39^ to illustrate the *realistic* cooling efficiency of our design. For Gr/WSe_2_/Gr with *d* = 62 nm and *κ* = 0.048 W/m/K, we have *T*_*R*_ = 425 K and our design predicts a cooling efficiency of 26.11% of the Carnot efficiency with an optimal barrier *ϕ*′ ≈ 0 (Ohmic contact). For Gr/MoSe_2_/Gr with *d* = 70 nm and *κ* = 0.0847 W/m/K, we have *T*_*R*_ = 496 K and the cooling efficiency is 21.94% of the Carnot efficiency with an ohmic contact too. If the *T*_*R*_ can be engineered to be less than 300 K, the maximum efficiency will occur at some optimal values of *ϕ*′ = 0.05 to 0.3 volt, which are also within the current tunable range for Gr/WSe_2_ and Gr/MoSe_2_ contacts^24^,^40^.

For practical TE-based coolers used in various applications (e.g. air-conditioned car seats, and semiconductor laser cooling), the efficiency is less than 15% of the Carnot efficiency^41^. With the highest reported value of *ZT* = 2.4 for Bi_2_Te_3_/Sb_2_Te_3_ superlattice structure^6^, the efficiency will become 31.1% of the Carnot efficiency at the same temperature studied here. For practical applications, the refrigerator has to pump a heat flux of a few hundreds W/cm^2^. For our design, the pumped heat current is estimated by 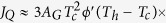 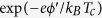 where *A*_*G*_ = 0.01158 A/cm^2^/K^3^. At *T*_*c*_ = 260 K, *T*_*h*_ = 300 K, and *ϕ*′ = 0.05 volt, the estimated cooling power is up to 500 W/cm^2^, which is larger than those of thin-film Bi_2_Te_3_-superlattice thermoelectric cooling devices^42^. At higher range of *T* = 400 to 500 K, our design will give about 1.7 to 3 kW/cm^2^.

### Power generator

When the current flow is from hot side to cold side (e.g. *T*_*h*_ = 400 K and *T*_*c*_ = 300 K). the device will behave as a power generator. This current flow via an external circuit is then extracted as the power output by harvesting the thermal energy from the heated graphene electrode (hot side). The maximal value of the power generation efficiency is calculated by


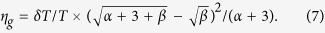


**Figure 4:**
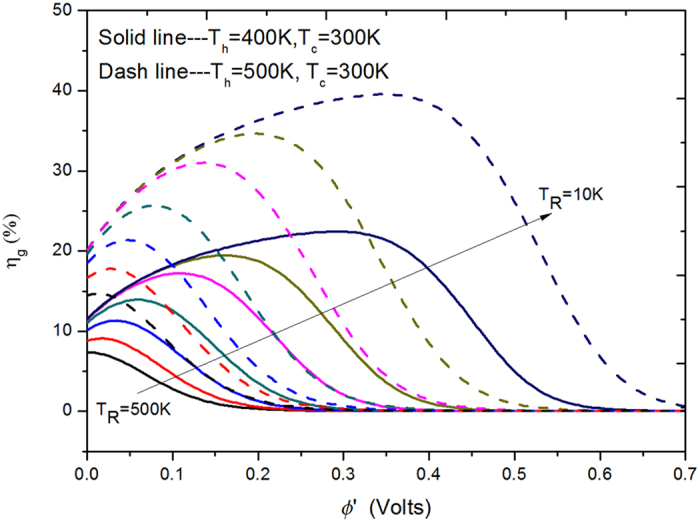
The power generation efficiency *η*_*g*_ as a function of barrier height *ϕ*′ at *T*_*c*_ = 300 K for *T*_*h*_ = 400 K (solid lines) and 500 K (dashed lines) from *T*_*R*_ [K] = 500, 400, 300, 200, 100, 50 and 10 (according to the arrow direction).

The calculated results are plotted in Fig. 4 as a function of *ϕ*′ for *T*_*R*_ = 500 K down to 10 K at a fixed *T*_*c*_ = 300 K for two heat sources: *T*_*h*_ = 400 K (solid lines) and 500 K (dashed lines). At *T*_*h*_ = 400 K, the efficiency is from about 8% to 20% for *T*_*R*_ = 500 K down to 10 K. Assuming *κ* = 0.08 W/m/K and *d* = 89 nm, this corresponds to *T*_*R*_ = 450 K and *η*_*g*_ is about 8% (with *ϕ*′ ≈ 0), which is comparable to or better than some of highly-efficient thermal harvesting devices, such as (a) a two-layer WSe_2_ TE-based device (*ZT* = 1.6) having a maximum efficiency of 6.5%^9^, (b) an electrochemical system for harvesting low-grade waste heat energy (<100 °C) with efficiency less than 8%^43^, and (c) a theoretical efficiency of 8% for *ZT* = 2.4 TE material at 400 K^1^. Note that the efficiency for a power generator is very sensitive to heat source temperature. When *T*_*h*_ is increased from 400 K (solid lines) to 500 K (dash lines), the efficiency increases by a factor of about 2 as shown in Fig. 4.

Based on the available experimental data^27^,^28^,^39^, we calculate (see Table 1) the efficiencies of harvesting heat at 400 K (cold side is kept at 300 K) for some practical design parameters: (a) Gr/MoS_2_/Gr with *d* = 50 nm, (b) Gr/WS_2_/Gr with *d* = 50 nm, (c) Gr/MoSe_2_/Gr with *d* = 70 nm, and (d) Gr/WSe_2_/Gr with *d* = 62 nm. The table shows that the operating range of *T*_*R*_ is from 850 K down to 428 K, with efficiency from 3.15% to 8.56%. The efficiency for both Gr/MoSe_2_/Gr and Gr/WSe_2_/Gr is about 7% to 8%, which is better than 3% to 4% generated by Gr/MoS_2_/Gr and Gr/WS_2_/Gr. Note that we only use *T*_*h*_ = 400 K as an example in the table. For other heat source temperatures *T*_*h*_ (with *T*_*c*_ = 300 K), the efficiency of the energy harvesting using these common vdW heterostructures can be estimated based on the fitted equation determining the cross-plane thermal conductivity as a function of temperature by *κ* = *a* + *bT* + *cT*^2^ + *dT*^3^. where *a, b, c* and *d* are the fitting parameters, and they can be found in the Supplementary materials (see S2).

## Discussions

In our model, the tunneling of low-energy electrons through the Schottky barrier at the interface will become important if the width of the barrier is small^35^. This consideration imposes a lower limit to the layer thickness *d* in our design to ensure that the injection of the electrons from the graphene electrode across the barrier is governed by the over-barrier process (thermionic emission) as assumed in the model. This minimal *d* may be estimated by using 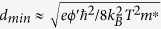, with m^*^ being the effective electron mass of the barrier layers^44^, which gives 0.845*m*_*e*_, 0.776*m*_*e*_, 0.665*m*_*e*_ 0.643*m*_*e*_, respectively, for MoS_2_, MoSe_2_, WS_2_ and WSe_2_. For Gr/WS_2_/Gr, it was reported that thermionic emission will be dominant for 5 layers or more^35^. For Gr/WSe_2_/Gr and Gr/MoSe_2_/Gr, the minimal thickness is estimated to be, respectively 5 and 4 nm based on Simmons model^45^.

For few-layer TMDs (MoS_2_, MoSe_2_ WS_2_ and WSe_2_), their cross-plane lattice thermal conductivity is very small compared to traditional TE materials, which is in the range of 0.01 to 0.1 W/m/K due to the localized lattice vibrations or the disorder within the TMDs^27^,^28^,^29^,^39^. It was claimed that the reduction is also valid for other reassembled TMDs^27^, and thus considering *d* = 50 nm and *κ* = 0.01 W/m/K, we have *T*_*R*_ around 272.6 K, which implies that it is positive to realize the calculated efficiencies of about 10% in harvesting waste heat at 400 K or 20% at 500 K as shown in Fig. 4 for *T*_*R*_ = 300 K.

As *T*_*R*_ scales as (*κ*/*d*)^1/3^, to further reduce *T*_*R*_ down to 100 K or even 10 K, the lattice cross-plane thermal conductivity *κ* must be reduced for a fixed thickness *d*. Further reducing *d* will not help as it will also promote the unwanted tunneling effect mentioned above. Thus, we conclude that our design will require vdW structure with low cross-plane thermal conductivity and reasonable thickness to function as a high-efficiency solid state thermionic device. A recent work for WSe_2_^46^ may have presented such a solution in increasing spacing of the layers, and stacking disorder, and it is possible have a further reduction in the cross-plane lattice thermal conductivity without reducing *d*. Another method to reduce the cross-plane thermal conductivity is by using superlattice composed of WSe_2_ and MoSe_2_. We speculate that a precise control of superlattice period thickness will be able to lead to a much lower cross-plane thermal conductivity together with fine-engineering thermal boundary resistance between different layers. If such extremely low cross-plane thermal conductivity can be realized experimentally, it is possible to have very high efficiency energy harvesting as indicated in Fig. 4.

**Figure 5:**
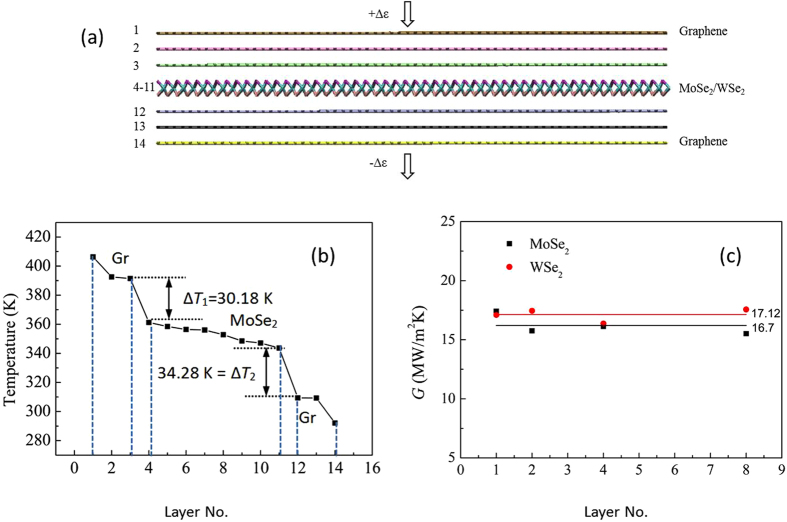
(**a**) Molecular dynamics model of interface thermal conductance of Gr/MoSe_2_/Gr. (**b**) The temperature distribution across the Gr/MoSe_2_/Gr vdW structure. Dotted lines are drawn to indicate the number of layers. (**c**) The interface thermal conductance (or Kapitza conductance) for Gr/MoSe_2_/Gr and Gr/WSe_2_/Gr as a function of the number of layers.

As mentioned before, the interface resistance between graphene and the TMDs has been neglected in our model. To justify this assumption, a Molecular dynamics simulation has been done. The simulation details can be found in the section of Method and Supplementary Materials (see S1). In Fig. 5a, multiple layers of MoSe_2_ (or WSe_2_) are sandwiched by few-layers graphene with three layers on top and three at the bottom. The temperature distribution along the cross-plane direction of the hybrid multilayer structure, with an imposed heat flux *J*_*Q*_ = 0.5 GW/m^2^ on the top graphene layer, is demonstrated in Fig. 5b. Note that small temperature drops near the heat source and heat sink are mainly induced by the artificial temperature control. From Fig. 5b, we see large temperature reduction Δ*T*_1_ = 30 *K* and Δ*T*_2_ = 34 *K* occurring at the two interfaces between graphene and MoSe_2_, respectively at the interface between layers 3 and 4, and also between layers 11 and 12. In comparison, the total temperature drop is about 2.8 K per layer for the eight layers of MoSe_2_ (one order of magnitude lower). The thermal conductance (or Kapitza conductance) for these two interfaces is about *G* = *J*/Δ*T* = 16.56 and 14.59 MW/m^2^/K, respectively. Note that the difference of *G* at the upper and the lower interface can be attributed to the temperature dependence of *G*^47^,^48^. These two values are larger than the cross-plane thermal conductance of a few-layer MoSe_2_ by one to two orders of magnitude using the experiment data^39^. Similar calculation has been repeated for WSe_2_, which shows similar results (not shown). In Fig. 5c, we show that the interface thermal conductance for WSe_2_ and MoSe_2_ is almost independent of the numbers of layers, and the average value is about *G* = 16.7 MW/m^2^/K for Gr/MoSe_2_/Gr and 17.12 MW/m^2^/K for Gr/WSe_2_/Gr. Surprisingly, we find that our calculated interface conductance across graphene and MoSe_2_ interface is very close to 25 MW/m^2^/K measured for graphene contact interface^48^. From these findings, it is important to note that it is valid to consider the layer’s resistance due to 2D TMDs in this paper as the first approximation. Subsequent improvement can be pursued through comparison with experimental verification (from other groups) on our calculated efficiency shown in Table 1 below. It is worth to mention that the calculated temperature gradient (around 50 K) across eight layers of MoSe_2_ (or WSe_2_) is consistent with the previous experiment^33^. So it is reasonable to believe that more significant temperature gradient across more layers of MoSe_2_ (or WSe_2_) can be established. Compared with larger temperature drop across Gr/TMDs/Gr structure, the little temperature drop across Graphene layers can be ignored. In other words, the cross-plane thermal conductivity of graphene layers is much higher than that of TMDs. Therefore solid thermionic converter based on Gr/TMDs/Gr structure has better performance than pure graphene layers (Gr/Gr/Gr). In the Table 1, we study some well-known TMDs (MoS_2_, MoSe_2_, WS_2_ and WSe_2_) using the reported experimental parameters, it is found that MoSe_2_ and WSe_2_ are better candidates. They are able to harvest waste heat at 400 K with about 7% to 8% in efficiency. To further increase the efficiency of a power generator, the current model can be also extended to include the contribution from solar energy and other thermal effects^49^,^50^,^51^. As a refrigerator, the cooling efficiency is about 0.22 to 0.26 of the Carnot efficiency for a temperature difference of 40 K between 260 and 300 K. Note that the effects of finite electrical conductivity and thermal conductivity in the lateral direction on the proposed device’s performance are not yet considered. The treatment of full model requires solving the coupling of Schrodinger and Dirac system, which is beyond the scope of this paper.

Similar to conventional TE devices, thermionic devices have lot of advantages, such as no moving parts, no noise, high reliability, long service time and so on. These features enables many possible applications for thermionic power generation and thermionic cooling. The most promising application for TIC is wasted heat recovery from vehicles to improve fuel economy. Other potential applications include harvesting industrial waste heat (e.g. steel rolling mill, cement, glass manufacture plant, etc.) and domestic heat (e.g. water heater). for electricity or charging batteries. While for thermionic cooling, most possible application is on-chip cooling of nanoelectronics devices. But the application can be extended to integrate with targeted devices to maintain the low-temperature environment for semiconductor laser, medical and scientific equipments. Finally, the vertical transport of charge transport at the graphene-semiconductor interface remains interesting. Modified Schottky models have been formulated to study the inhomogeneous of Schottky barrier at the interface^52^ and also the smooth transition between the T^3^ and T^2^ temperature scaling^53^.

## Methods

The interlayer distances between the graphene and MoSe_2_ (or WSe_2_) are set to 3.35 Å which is interlayer distance of bulk graphite. The interlayer distance between the MoSe_2_ (or WSe_2_) layers is 3.11 Å (or 3.14 Å) according to previous first-principle calculation^54^. The in-plane dimensions of the layer considered here are 69 *Å* × 70 *Å*. Periodic boundary conditions are applied along the in-plane directions and free boundary conditions are applied along the cross-plane direction of heterostructure. In the simulation, the initial configuration is equilibrated by using the constant volume and temperature (NVT) ensemble at a temperature *T* for 50 ps with a time step Δ*t* = 0.5 fs. Upon realization of the equilibrium state, the system is switched to the constant volume and energy (NVE) ensemble to maintain the energy conservation condition. A constant heat flux is then imposed into the system at each time step by adding a small amount of heat Δ*ε* into the upmost graphene layer (layer 1). In doing so, we reduce the same amount of energy from the graphene layer at the bottom (layer 14). The simulation is conducted until a stable temperature gradient is established along the heat flux direction. For a hybrid system consisting of different interfaces, a temperature drop Δ*T*_*ln*_ at the interface is usually developed, which gives a measurement of the interface thermal conductance or the Kapitza conductance *G* = *J*/Δ*T*_*ln*_, where *J* = Δ*ε*/*A*Δ*t* with *A* denoting the cross-section area.

## Additional Information

**How to cite this article:** Liang, S.-J. *et al*. Thermionic Energy Conversion Based on Graphene van der Waals Heterostructures. *Sci. Rep.* **7**, 46211; doi: 10.1038/srep46211 (2017).

**Publisher's note:** Springer Nature remains neutral with regard to jurisdictional claims in published maps and institutional affiliations.

## Supplementary Material

Supplementary Information

## Figures and Tables

**Figure 1:**
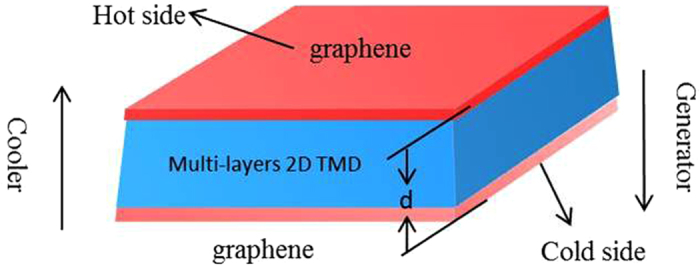
A novel design of solid-state vdW heterostructure-based thermionic devices. This structure is composed of multilayers of 2D TMDs material (such as MoS_2_, WS_2_, MoSe_2_ and WSe_2_) with thickness *d* sandwiched between two graphene electrodes. The top graphene electrode is attached to a heat source at temperature *T*_*h*_ and the bottom one is maintained at room temperature (*T*_*c*_ = 300 K) when functioning as a power generator. The electrons (or holes) are thermally-excited and ballistically transported over the Schottky barrier formed at the interface between the graphene electrodes and 2D vdW structure. As a refrigerator, the cold side is reduced to *T*_*c*_ = 260 K through removing the high-energy electrons or holes in the graphene electrode and the heat is carried by the hot electrons (holes) to the hot electrode biased at room temperature *T*_*h*_ = 300 K.

**Figure 2:**
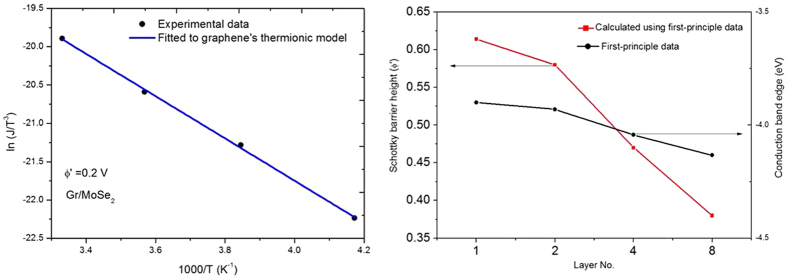
(**a**) The comparison of the thermionic current for a Gr/MoSe_2_ contact between the calculation and experiment results^24^: *ln(J*/*T *^3^) as a function of 1000/*T* with an effective barrier height of *ϕ*′ = 0.2 V for 44 layers of MoSe_2_. (**b**) Schottky barrier height (*ϕ*′) [left y-axis] of graphene/MoSe_2_ contact decreases with increasing layer number of MoSe_2_, which is calculated^37^ based on the conduction band edge of MoSe_2_ [right y-axis].

**Table 1:** The designed parameters: *T*_*R*_, optimal effective barrier height *ϕ*′ and the calculated efficiency *η*_*g*_ of the proposed power generator operating at *T*_*h*_ = 400 K and *T*_*c*_ = 300 K based on the experimentally-measured cross-plane thermal conductivity of different TMDs materials at the temperature of 300 K [See S2: Supplementary materials]: (a) 50-nm of Gr/MoS_2_/Gr^28^, (b) 50-nm of Gr/WS_2_/Gr^28^, (c) 70-nm of Gr/MoSe_2_/Gr^27^,^39^ and (d) 62-nm of Gr/WSe_2_Gr/27,39

System	*d* [nm]	*κ* for [W/m/K]	Experiment SBH [V]	*T*_*R*_ [K]	Optimal SBH [V]	Max *η*_*g*_ [%]
G/MoS_2_	50	0.3	0 to 0.11	847	0	3.15
G/WS_2_	50	0.2125	0 to 0.37	758	0	3.93
G/MoSe_2_	70	0.089	0 to 0.4	501	0.005	7.28
G/WSe_2_	62	0.048	0 to 0.44	428	0.02	8.56
