# Supplementary material for: Thermionic Energy Conversion Based on Graphene van der Waals Heterostructures
Source: Sci Rep. 2017 Apr 7;7:46211. doi: 10.1038/srep46211 (PMC5384245; doi:10.1038/srep46211)
Supplement: Supplementary Information [file srep46211-s1.doc]

| Supplementary Material  **Thermionic Energy Conversion Based on Graphene van der Waals Heterostructures** |
| --- |
| Shi-Jun Liang^1^, Bo Liu^2^, Wei Hu^3^, Kun Zhou^2^ and L. K. Ang^1^ (**🖂**).  *^1^SUTD-MIT International Design Center (IDC), Singapore University of Technology and Design (SUTD), 8 Somapah road 487372 Singapore*  *^2^School of Mechanical and Aerospace Engineering, Nanyang Technological University, 50 Nanyang Avenue,* *639798,Singapore*  *^3^Computational Research Division, Lawrence Berkeley National Laboratory, Berkeley, CA 94720, USA* |

**Table of contents**

**S1. Molecular dynamics simulation of thermal conductance through the interface of graphene and MoSe_2_ or WSe_2_ layer**

**S2. Temperature dependence of thermal conductivity**

**S3. Schottky Barrier Height of graphene/TMD contact via First-Principle Calculation**

S1. **Molecular dynamics simulation of thermal conductance through the interface of graphene and TMD**

1. **Potential functions**

The inter-atomic interactions within each graphene layer are described by the optimized Tersoff potential . The non-bonded interactions between graphene and MoSe_2_ (or WSe_2_) are described by the 12-6 Lennard-Jones (LJ) potential: $V(r)=4\varepsilon\left[\left(\frac{\sigma}{r}\right)^{12}-\left(\frac{\sigma}{r}\right)^{6}\right]$, with the corresponding LJ parameters adopted from the UFF force filed , which are listed in Table 1. For MoSe_2_ and WSe_2_, both the bonded and non-bonded interactions are described by the LJ potential. The intra-sheet covalent bonding and inter-sheet van der Waals bonding parameters for WSe_2_ have been reported in previous study and were obtained by fitting them to the WSe_2_ lattice parameters and its elastic constants. To have a better comparison between MoSe_2_ and WSe_2_, the same methodology has been adopted to obtain the LJ parameters for MoSe_2_ in the present study. The LJ parameters for the bonded and non-bonded interactions inside and between the MoSe_2_ (or WSe_2_) layers are listed in Tables 2 and 3, respectively. Due to the similarity between MoSe_2_ and WSe_2_, the LJ parameters for the van der Waals interaction between the MoSe_2_ and WSe_2_ sheets are set to be the average values between MoSe_2_ and WSe_2_.

Table 1 LJ potential parameters for the atomic interactions between graphene layers and those between graphene and MoSe_2_ or WSe_2_

| **Atomic Pairs** | ***σ* (Å)** | ***ε* (meV)** |
| --- | --- | --- |
| **C-C** | 3.44 | 4.6 |
| **C-Mo** | 3.08 | 3.34 |
| **C-W** | 3.09 | 3.65 |
| **C-Se** | 3.60 | 7.61 |

Table 2 LJ potential parameters of the bonded atomic interactions for individual MoSe_2_ and WSe_2_ sheets

| **Material** | **Atomic pairs** | **σ (Å)** | ***ε* (meV)** |
| --- | --- | --- | --- |
| **MoSe_2_** | Mo-Mo | 2.97 | 320.5 |
|  | Se-Se (same atomic plane) | 2.97 | 320.5 |
|  | Se-Se (different atomic plane) | 2.30 | 320.5 |
| **WSe_2_** | W-W | 2.94 | 341.7 |
|  | Se-Se (same atomic plane) | 2.94 | 341.7 |
|  | Se-Se (different atomic plane) | 2.28 | 341.7 |

Table 3 LJ potential parameters of the non-bonded atomic interactions between different MoSe2 or WSe2 sheets

| **Material pairs** | **σ (Å)** | ***ε* (meV)** |
| --- | --- | --- |
| **MoSe_2_-MoSe_2_** | 3.45 | 22.5 |
| **WSe_2_-WSe_2_** | 3.40 | 19.9 |
| **WSe_2_-MoSe_2_** | 3.42 | 21.2 |

**S2. Temperature dependence of thermal conductivity**

In Fig. 1, the temperature dependence up to 300 K of the cross plane thermal conductivity $\kappa$[in unit of W/m/K] of WSe_2_ and MoSe_2_ is shown, where the symbols representing experimental data [5,6] and solid lines are fitted to these experimental data using cubic polynomial: $\kappa(T)=a+bT+cT^{2}+dT^{3}$(see table 4 below). Using these fitted equations, the thermal conductivity can be determine for any temperature, which is convenient for experimentalists to estimate the maximum efficiency of solid thermionic cooler or power generator (see main manuscript).

Table 4 The fitting constants for $\kappa(T)=a+bT+cT^{2}+dT^{3}$

|  | ***a*** | ***b*** | ***c*** | ***d*** |
| --- | --- | --- | --- | --- |
| **24 nm WSe_2_** | -0.021988 | 9.358 × 10^-4^ | 3.81 × 10^-6^ | 5.35 × 10^-9^ |
| **70 nm MoSe_2_** | -0.02585 | 1.2595 × 10^-3^ | -4.946 × 10^-6^ | 6.716 × 10^-9^ |
| **62 nm MoSe_2_** | -0.0135 | 6.894 × 10^-4^ | -2.7086 × 10^-6^ | 3.6777 × 10^-9^ |


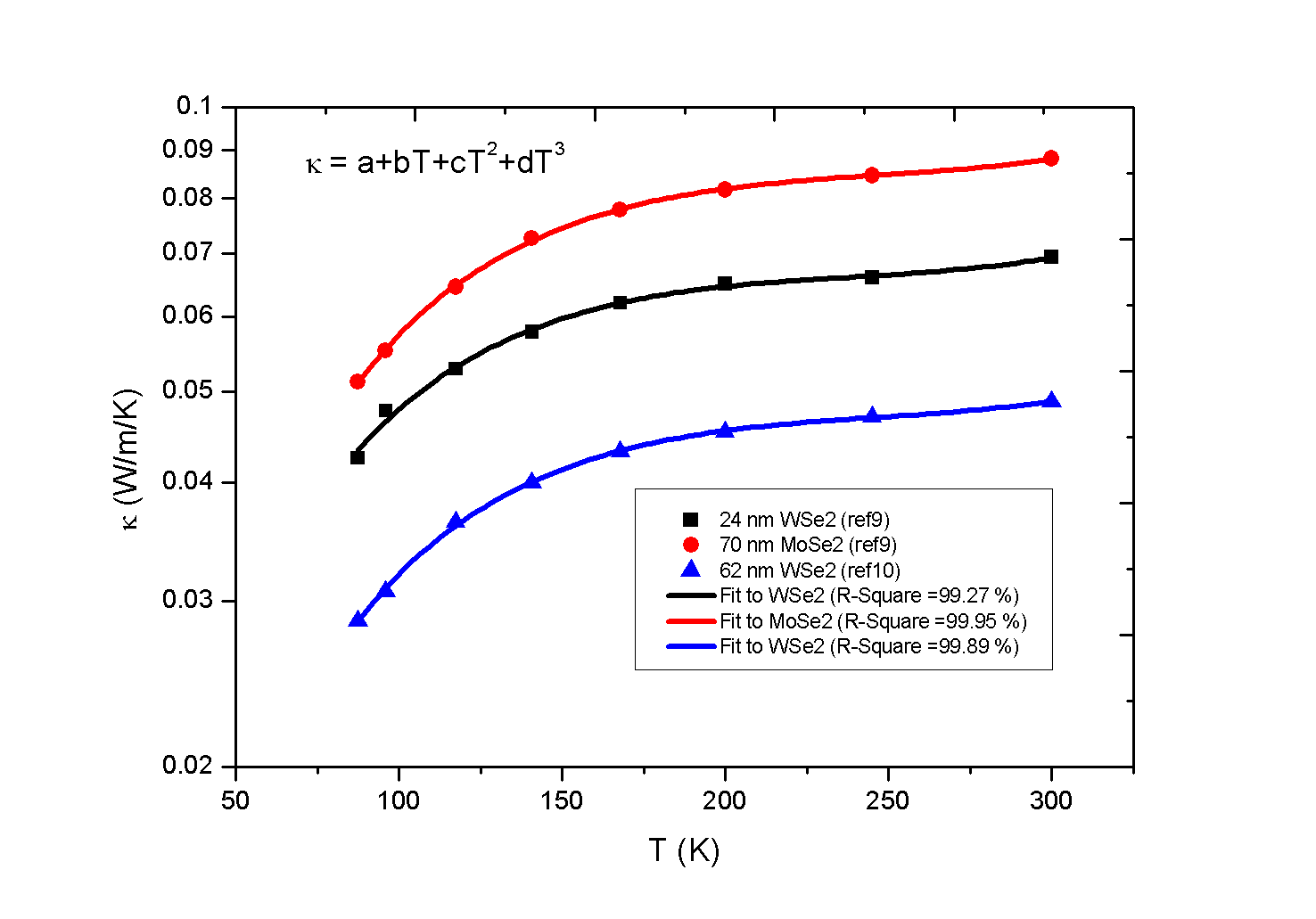


Fig. 2 Experimental thermal conductivity (symbols) of WSe_2_ and MoSe_2_ as a function of temperature and fittings to $\kappa=a+bT+cT^{2}+dT^{3}$for different materials (WSe2 and MoSe2).

**S3. Schottky Barrier Height of graphene/TMD contact via First-Principle Calculation**

First-principles calculations are based on the density functional theory (DFT) implemented in the VASP package [7]. We adopt the generalized gradient approximation (GGA) [8] for the exchange correlation functional. The non-empirical van der Waals density functional (vdW-DF) scheme proposed by Dion et al [9] is chosen due to its good description of long-range vdW interactions. The energy cutoff is set to be 500 eV. The surface Brillouin zone is sampled with a 4×4 regular mesh and 120 k points are used for calculating the tiny band gaps at the Dirac point graphene in the hybrid nanocomposite supercell. All the geometry structures are fully relaxed until energy and forces are converged to 10^-5^eV and 0.01 eV/Å, respectively. The relevant parameters for calculating band structure are tabulated in Table 5.

First of all, the band structures for graphene (5x5, 4x4), MoS_2_, WS_2_, MoSe_2_ and WSe_2_ have been calculated using VASP to test whether our calculation approaches are reasonable as shown in Fig. 3. The calculated band structure for contact between graphene (Gr) and one layer of different transition metal dichalcogenides (TMDs) is shown in the Fig. 4. The Schottky barrier height (n-SBH or p-SBH) is extracted for each contact system through the difference between lowest conduction (valence) band edge and Fermi level. Note that we set all Fermi level to be zero in the calculation (see green lines). Table 5 show the simulated information and parameters used for the optimized structure.


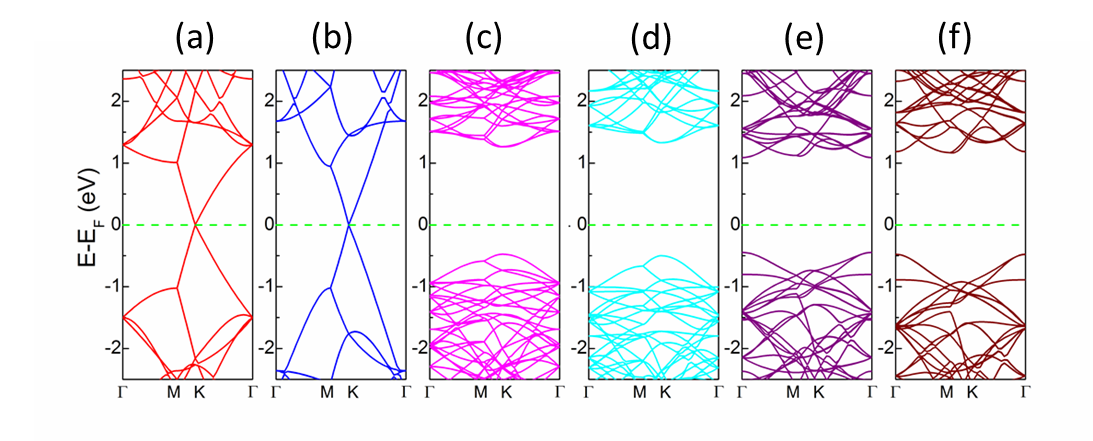


Fig. 3 Calculated band structures for (a) graphene (5×5), (b) graphene (4×4), (c) MoS_2_, (d) WS_2_, (e) MoSe_2_, ( f ) WSe_2_.


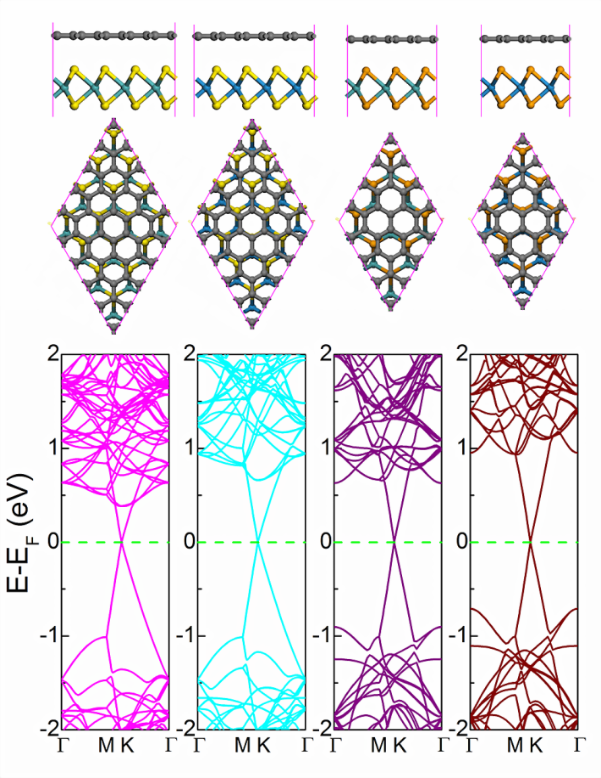


Fig. 4 (from left to right) Optimized structures and calculated band structures for Gr/MoS_2_, Gr/WS_2_, Gr/MoSe_2_ and Gr/WSe_2_. The upper panel shows the side and top views of graphene and TMDs contact.

Table 5 The interlayer distance and binding energy for different optimized structure

| **System** | **Deq (Å)** | **E_b_ (meV/atom)** |
| --- | --- | --- |
| **Gr/MoS_2_** | 3.3707 | -62.27 |
| **Gr/WS_2_** | 3.3864 | -62.28 |
| **Gr/MoSe_2_** | 3.4794 | -57.81 |
| **Gr/WSe_2_** | 3.4819 | -57.68 |

**Reference**

1. Lindsay, L., and D. A. Broido. "Optimized Tersoff and Brenner empirical potential parameters for lattice dynamics and phonon thermal transport in carbon nanotubes and graphene." Physical Review B 81.20 (2010): 205441.

2. Huang X, Tan C, Yin Z, Zhang H. Hybrid Nanostructures Based on Two-Dimensional Nanomaterials. Advanced Materials. 2014;26:2185-204. 10.1002/adma.201304964.

3. Rappé, Anthony K., et al. "UFF, a full periodic table force field for molecular mechanics and molecular dynamics simulations." Journal of the American Chemical Society 114.25 (1992): 10024-10035.

4. Shen, Meng, and Pawel Keblinski. "Ballistic vs. diffusive heat transfer across nanoscopic films of layered crystals." Journal of Applied Physics 115.14 (2014): 144310.

5. Chiritescu, Catalin, David G. Cahill, Ngoc Nguyen, David Johnson, Arun Bodapati, Pawel Keblinski, and Paul Zschack. "Ultralow thermal conductivity in disordered, layered WSe2 crystals." Science 315, no. 5810 (2007): 351-353.

6. Chiritescu, C. Ultralow thermal conductivity in layered disordered crystaline materials. Ph.D Thesis, University of Illinois at Urbana-Champaign (2010).

7. Kresse, Georg, and Jürgen Hafner. "Ab initio molecular dynamics for liquid metals." Physical Review B 47.1 (1993): 558.

8. Perdew, John P., Kieron Burke, and Matthias Ernzerhof. "Generalized gradient approximation made simple." Physical review letters 77.18 (1996) 3865.

9. Dion, Max, et al. "Van der Waals density functional for general geometries." Physical review letters 92.24 (2004) 246401.
